# Mortality on extreme heat days using official thresholds in Spain: a multi-city time series analysis

**DOI:** 10.1186/1471-2458-12-133

**Published:** 2012-02-17

**Authors:** Aurelio Tobias, Ben Armstrong, Ines Zuza, Antonio Gasparrini, Cristina Linares, Julio Diaz

**Affiliations:** 1 Institute of Environmental Assessment and Water Research (IDAEA), Spanish Council for Scientific Research (CSIC), Barcelona, Spain; 2 Department of Social and Environmental Health Research, London School of Hygiene and Tropical Medicine (LSHTM), London, UK; 3 Biomedical Research Foundation of the Gregorio Marañón University Hospital, Madrid, Spain; 4 National School of Public Health (ENS), Instituto de Salud Carlos III (ISCIII), Madrid, Spain; 5 National Centre for Epidemiology (CNE), Instituto de Salud Carlos III (ISCIII), Madrid, Spain

## Abstract

**Background:**

The 2003 heat wave had a high impact on mortality in Europe, which made necessary to develop heat health watch warning systems. In Spain this was carried-out by the Ministry of Health in 2004, being based on exceeding of city-specific simultaneous thresholds of minimum and maximum daily temperatures. The aim of this study is to assess effectiveness of the official thresholds established by the Ministry of Health for each provincial capital city, by quantifying and comparing the short-term effects of above-threshold days on total daily mortality.

**Methods:**

Total daily mortality and minimum and maximum temperatures for the 52 capitals of province in Spain were collected during summer months (June to September) for the study period 1995-2004. Data was analysed using GEE for Poisson regression. Relative Risk (RR) of total daily mortality was quantified for the current day of official thresholds exceeded.

**Results:**

The number of days in which the thresholds were exceeded show great inconsistency, with provinces with great number of exceeded days adjacent to provinces that did not exceed or rarely exceeded. The average overall excess risk of dying during an extreme heat day was about 25% (RR = 1.24; 95% confidence interval (CI) = [1.19-1.30]). Relative risks showed a significant heterogeneity between cities (I^2 ^= 54.9%). Western situation and low mean summer temperatures were associated with higher relative risks, suggesting thresholds may have been set too high in these areas.

**Conclusions:**

This study confirmed that extreme heat days have a considerable impact on total daily mortality in Spain. Official thresholds gave consistent relative risk in the large capital cities. However, in some other cities thresholds

## Background

According to the most recent World Health Organisation executive committee report, the 2003 summer heat wave caused a total of 44,000 excess deaths in Europe as a consequence of the extremely high temperatures recorded that summer [1]. Since that, many European countries have drawn up heat-surveillance systems to alert and prevent the negative effects of extreme heat temperatures on their health [2]. The Spanish Government, through their Ministry of Health, started implementing in 2004 the *"National plan for preventive actions against the effects of excess temperatures on health" *[3]. This Plan sets city-specific thresholds for extreme heat days during summer months (from June to September) for each of the 52 provincial capital cities. Although, extreme heat temperatures have been associated with an increase of mortality [4-7] and morbidity [8,9] in some Spanish cities, the health impact of extreme heat days established under the Ministry's official definition has not yet been assessed. Therefore, this could be considered the first national study conducted in Spain.

The objective of this study is to assess effectiveness of the official temperature thresholds established by the Ministry of Health for each provincial capital city in identifying high-risk days by quantifying short-term effects of above-threshold days on total daily mortality in each city over the ten years prior to the operation of the Plan. In particular we explored whether the implicit above-threshold mortality risk level has been similar across cities. We also explore determinants of variation in the city-specific risks.

## Methods

### Setting

Spain is the second largest country in Western Europe (504,030 km^2^) with a total population of 46 million people. Its mainland is bordered to the South and East by the Mediterranean Sea; to the North by France, and the Bay of Biscay; and to the North-west and West by the Atlantic Ocean and Portugal. Spanish territory also includes the Balearic Islands in the Mediterranean, the Canary Islands in the Atlantic Ocean off the African coast, and two autonomous cities in Northern Africa, Ceuta and Melilla. It is divided in 17 Autonomous Regions, each composed from one up to nine provinces, and each province has a capital city (Additional file 1 shows their location and Additional file 2 reports major geographic and socio-demographic characteristics for each provincial capital city).

Due to Spain's geographical situation and conditions, the climate is extremely diverse. It can be roughly divided into five main zones: Mediterranean climate extends along the southern and eastern coasts up to the Pyrenees; Semiarid Mediterranean climate in the South-east; Continental Mediterranean climate in the inland areas of the Peninsula; Oceanic climate in the North-west and the coastal strip near the Bay of Biscay; and a Subtropical climate in the Canary Islands [10].

### Mortality data

Data on total daily all-cause mortality, excluding accidents (International Classification of Diseases-9^th ^revision/ICD-9: 1-799), for the 52 capital cities were provide by the Spain National Institute of Statistics for summer months (from 1st June to 30th September) from 1995 to 2004.

### Temperature data and extreme heat days

Daily minimum and maximum temperatures (in C) for the 52 capital cities were collected from the Spain National Meteorology Agency for the same study period. In two capital cities, Palencia and Ceuta, daily temperatures were not available.

Using these data, city-specific extreme heat days were defined under the official thresholds established by the *"National Plan for Preventive Actions against the Effects of Excess Temperatures on Health" *of the Ministry of Health. This Plan sets city-specific thresholds as follows: *"The criteria have been established based on studies sponsored by the Ministry of Health, the observations made by the Autonomous Regions and the technical details of National Meteorology Agency. Extreme heat days are based on the 95^th ^percentile of the 20-year historical series of daily summer (from June to September) of minimum and maximum temperatures for each of the 52 cities, which are capitals of provinces. As exceptions, for the mild climate stations with low daily temperature variations (mainly sea areas), Northern and North-western Spain, the threshold for the maximum temperature correspond to the 95^th ^percentile of the series of absolute maximum temperatures of each summer. Similarly, in continental climate stations, the threshold considered for the minimum temperature corresponds to the 95^th ^percentile of the series of higher minimum temperatures of summer. The values obtained through these calculations have been rounded to nearest integer, because the error in predicting temperature maximum and minimum is about one Celsius degree. In capital cities where the threshold for minimum temperature were below 20 C and below 33 C for maximum temperature, these values have been assigned because minimum temperatures lower than 20 C and maximum lower than 33 C do not have consequences for the purposes of this Plan" *[3] (Author's translation). This means that original thresholds given by 95^th ^percentiles were revised upwards in cooler cities. The activation of this Plan occurs when both thresholds, for minimum and maximum daily temperatures, are simultaneously exceeded.

### Statistical analysis

Following methodology used in studies previously conducted in other European cities [2,11,12] the risk of mortality on extreme heat days was quantified using Generalised Estimating Equation models (GEE) with the number of daily deaths being assumed to follow a Poisson distribution. We modelled the marginal relationship between total daily mortality and a binary variable for extreme heat days in each capital city, assuming independence among summers and treating serial dependence of daily number of deaths within each summer as a nuisance parameter by specifying a first-order autoregressive correlation structure in the Poisson GEE model. Long-term trends were modelled using natural cubic splines of the variable time allowing one degree of freedom (df) for every 5-year of data. To allow for within summer seasonal variation not explained by extreme heat days, we fit natural cubic splines of day-in-year (4 df) constrained to be the same over all years.

A binary variable (0,1) was defined to identify extreme heat days for each capital city using the official thresholds, as previously described. To avoid inclusion in the baseline of days with temperatures just below the thresholds, which could still include some affected by heat, we used as a comparison category those days on which the 50^th ^percentile of both, minimum and maximum temperatures in summer months, was not simultaneously exceeded (i.e. days of below-median minimum and maximum temperature).

Overall estimates of the extreme heat relative risks were obtained by combining the city-specific estimates with a random-effects meta-analysis, when estimates between cities were heterogeneous [13]. To explore heterogeneity of effects we used random-effects meta-regression [14] to identify whether size of extreme heat relative risk was associated with city-specific geographic, socio-demographic and climatic characteristics.

All analyses were done using Stata 11.2 (StataCorp, College Station, TX, 2010).

## Results

### Extreme heat days

**Figure 1:**
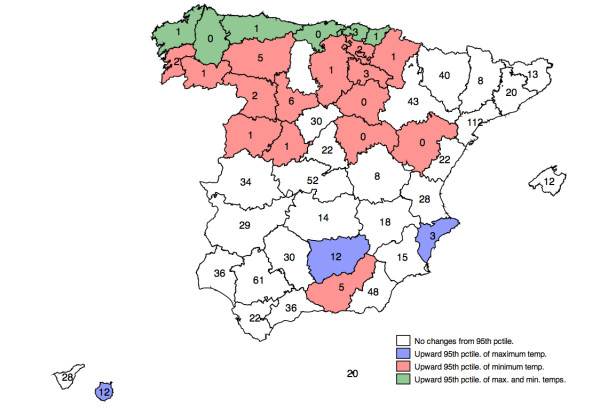
**Geographical distribution of total number on days when official thresholds for minimum and maximum temperatures were exceeded, identifying if exceptions to the use of 95th percentile had been used**.

Table 1 shows the distribution of daily temperature and the number of days when official thresholds for extreme heat days were exceeded for the study period and Figure 1 shows its geographical distribution identifying if exceptions to the use of 95th percentile had been used. These range from 0 (in Teruel, Santander, Guadalajara, Soria and Lugo) to 112 days (in Tarragona). In half of these cities the threshold was exceeded less than 10 days, while a fourth part of the cities was exceeded between 10 and 30 days, otherwise in more than 30 days. The year 2003 was when the limit was exceeded in a greater number of capital cities (35), followed by the year 1995 (29 cities), whilst the years where the threshold was exceeded by a smaller number of capital cities were in 1997 and 1996 (6 and 14, respectively).

**Table 1:** Description of minimum and maximum temperatures, official thresholds of the National plan, and total number of days when those thresholds were simultaneously exceeded (num. days)

		Daily temperatures (°C)						Official		
	**Meteorological**	**Minimum**			**Maximum**			**thresholds**		**num**

**City (capital)**	**Station**	**mean**	**(sd)**	**P95**	**mean**	**(sd)**	**P95**	**Min**	**Max**	**days**

**Almería**	Aeropuerto	21.0	(2.4)	24.8	29.4	(3.3)	35.6	24	35	48

**Cádiz**	Cortadura	20.8	(2.0)	24.2	26.4	(3.2)	32.4	24	33	22

**Córdoba**	Aeropuerto	18.0	(2.5)	22.2	34.3	(4.3)	41.0	22	41	30

**Granada**	Base Aérea	16.0	(2.8)	20.6	31.8	(4.4)	38.2	23	39	5

**Huelva**	Ronda Este	18.1	(2.2)	21.8	30.7	(3.9)	37.6	22	37	36

**Jaén**	Cerro de los Lirios	19.4	(3.3)	24.9	30.4	(4.5)	37.4	25	39	12

**Málaga**	Aeropuerto	19.8	(2.4)	23.8	29.6	(3.4)	36.4	23	36	36

**Sevilla**	Aeropuerto	19.6	(2.4)	23.5	33.7	(4.0)	40.3	22	40	61

**Huesca**	Monflorite	15.1	(3.6)	20.6	29.0	(4.4)	36.0	20	36	40

**Teruel**	Teruel	11.7	(3.1)	16.0	28.6	(4.6)	35.3	20	35	0

**Zaragoza**	Aeropuerto	16.9	(2.9)	21.2	30.2	(4.6)	37.5	21	37	43

**Oviedo**	El Cristo	13.8	(2.4)	17.5	22.4	(3.5)	28.5	20	33	1

**P. Mallorca**	Centro Meteorológico	17.6	(2.9)	22.1	29.9	(3.3)	35.0	22	35	12

**Las Palmas**	Telde - Aeropuerto G. Canaria	21.2	(1.4)	23.1	27.0	(1.8)	29.8	23	33	12

**Tenerife**	Santa Cruz de Tenerife	21.5	(1.6)	23.9	28.6	(2.1)	32.2	23	33	28

**Santander**	Parayas - Aeropuerto	15.4	(2.5)	19.4	23.1	(2.9)	28.0	22	35	0

**Albacete**	Los Llanos - Base Aérea	15.3	(3.0)	19.6	30.6	(4.4)	36.8	20	37	18

**Ciudad Real**	Escuela Magisterio	17.5	(3.2)	22.6	31.9	(4.7)	38.6	22	39	14

**Cuenca**	Cuenca	14.2	(3.4)	19.6	28.7	(4.5)	35.1	21	35	8

**Guadalajara**	El Serranillo	12.0	(3.4)	17.0	31.2	(4.7)	38.0	21	37	0

**Toledo**	Buenavista	17.2	(3.3)	22.4	32.1	(4.6)	38.8	22	38	52

**Ávila**	Observatorio	11.9	(3.6)	17.7	26.1	(4.7)	33.0	22	33	1

**Burgos**	Villafria	10.6	(3.2)	15.4	26.0	(5.2)	34.4	20	33	1

**León**	Virgen del Camino	11.3	(3.3)	16.6	25.1	(4.8)	32.4	20	33	5

**Palencia**	NA									

**Salamanca**	Matacán	11.7	(2.9)	16.0	27.8	(4.7)	34.8	20	35	1

**Segovia**	Observatorio	13.2	(3.8)	19.6	27.2	(4.9)	34.3	20	34	30

**Soria**	Observatorio	11.2	(3.0)	15.8	26.2	(5.1)	33.6	20	34	0

**Valladolid**	Observatorio	13.0	(3.1)	18.0	28.4	(4.8)	35.8	21	36	6

**Zamora**	Observatorio	13.7	(3.1)	18.6	28.2	(4.7)	35.6	22	35	2

**Barcelona**	Aeropuerto El Prat	19.0	(2.7)	23.1	26.8	(2.9)	31.6	22	33	20

**Girona**	Aeropuerto Girona-Costa Brava	15.4	(2.9)	19.5	28.4	(3.9)	34.4	20	34	13

**Lleida**	Observatori 2	16.1	(3.2)	20.5	30.6	(4.09	36.8	21	37	8

**Tarragona**	Observatorio del Ebro	19.1	(2.6)	23.0	30.9	(3.4)	35.6	22	33	112

**Ceuta**	NA									

**Melilla**	Melilla	21.1	(2.0)	24.2	27.5	(2.7)	32.4	24	33	20

**Badajoz**	Base Aérea	16.5	(2.6)	21.0	32.7	(4.4)	39.8	21	40	29

**Cáceres**	Carretera Trujillo	17.3	(3.2)	22.8	31.3	(4.7)	38.5	23	38	34

**A Coruña**	Estación Completa	15.7	(1.7)	18.4	22.1	(2.69	26.7	20	33	1

**Lugo**	Aeródromo	11.0	(3.0)	15.4	23.6	(4.3)	31.5	20	33	0

**Ourense**	Granxa Deputación	13.8	(2.9)	18.4	29.1	(4.8)	36.6	21	37	1

**Pontevedra**	Mourente	14.4	(2.3)	18.0	24.7	(4.0)	32.0	22	33	2

**Logroño**	Agoncillo	14.4	(3.0)	19.0	28.2	(5.0)	36.4	22	36	3

**Madrid**	Madrid - Retiro	17.4	(3.3)	22.6	29.3	(4.4)	35.8	21	37	22

**Murcia**	Murcia	19.7	(2.4)	23.3	32.3	(3.1)	37.4	22	38	15

**Pamplona**	Noain	13.6	(3.1)	18.3	26.6	(5.4)	35.6	22	36	1

**Bilbao**	Aeropuerto	14.8	(2.9)	19.4	24.6	(4.1)	32.5	21	37	3

**San Sebastián**	Igueldo	15.6	(2.4)	19.4	21.5	(3.8)	29.2	22	36	1

**Vitoria**	Aeropuerto Foronda	11.5	(3.5)	16.8	24.6	(5.1)	33.3	20	34	2

**Alicante**	Ciudad Jardín	20.1	(2.3)	23.6	29.5	(2.4)	33.5	23	35	3

**Castellón**	Alzamora	19.8	(2.5)	23.4	29.2	(2.6)	33.0	23	33	22

**Valencia**	Valencia	20.5	(2.4)	24.1	29.5	(2.7)	34.0	23	34	28

NA: data not available

### Risk of mortality

**Figure 2:**
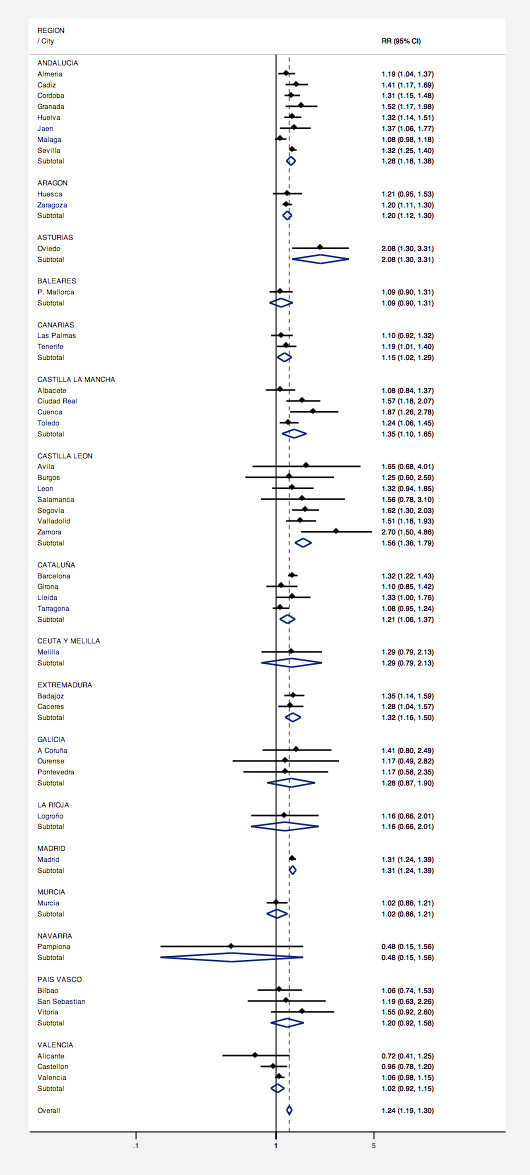
**Relative risk (RR), and its 95% confidence interval (95%CI), of dying on days when official thresholds for minimum and maximum temperatures were exceeded (black dots denote city-specific estimates, blue diamonds denote Autonomous Region estimates, dashed red line denotes overall estimate)**.

Figure 2 shows the relative risk (RR) of dying on days on which the official thresholds, for both minimum and maximum temperatures, were exceeded with respect to a baseline comprising those days on which the maximum and minimum temperatures were simultaneously below the 50^th ^percentile. The overall excess risk of dying during an extreme heat day was about 25% (RR = 1.24; 95% confidence interval (CI) = [1.19-1.30]).

**Figure 3:**
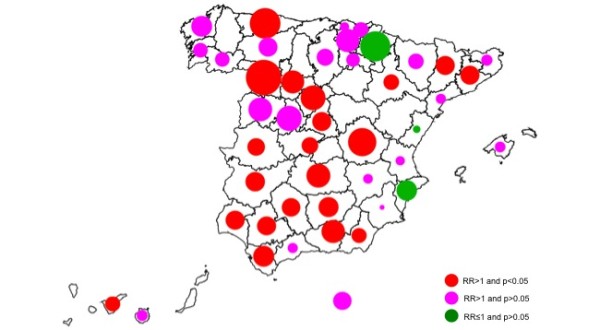
**Geographical distribution of the risk of dying (symbol size proportional to relative risk) on days when official thresholds for minimum and maximum temperatures were exceeded (red circles denote statistically significant risks of dying (*p *< 0.05), magenta circles denote non-statistically significant risk of dying, and green circles denote non-statistically significant protective effect)**.

Estimates between cites showed a significant heterogeneity (I^2 ^= 54.9%, p < 0.001). The highest city-specific risks were found for Zamora (RR = 2.70; 95%CI = [1.50-4.86]), Oviedo (RR = 2.08; 95%CI = [1.30-3.31]) and Cuenca (RR = 1.87; 95%CI = [1.26-2.78]). The largest cities showed similar risk estimates, close to 30% (Madrid: RR = 1.31; 95%CI = [1.24-1.39], Barcelona RR = 1.32; 95%CI = [1.22-1.43], and Seville RR = 1.32; 95%CI = [1.25-1.40]). The geographical pattern (Figure 3) seems to be a trend from East, with lower risks, to West, with higher risks. Comparing mean risk estimates between Autonomous Regions showed higher values for central regions and lower values for those in the Mediterranean area.

### Exploring heterogeneity

**Figure 4:**
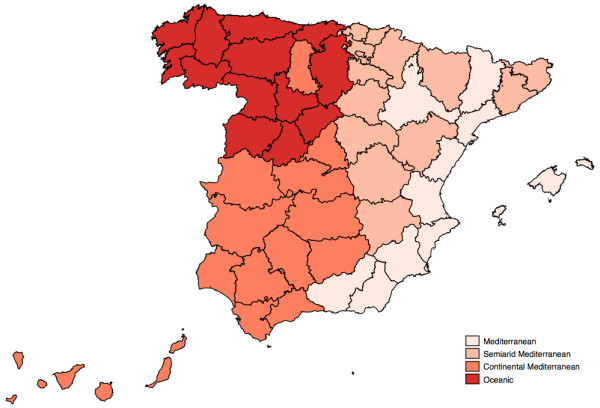
**Geographical distribution of variation in risk of mortality due to extreme heat days due to latitude and mean temperature**.

To explain the variation in risk of mortality due to above-threshold days across cities we considered geographic (latitude, longitude, and altitude), socio-demographic (population density, proportion of people older than 65 years, and per capita personal income) and climatic (mean temperature, mean humidity, and total hours of sunshine in summer) characteristics for each capital city. Although many of these were significant determinants of extreme heat risk when considered singly, in a multiple meta-regression we found that a model including only latitude and mean temperature in summer explained most of the heterogeneity between city-specific risk estimates (residual heterogeneity, I^2 ^= 28.3%), leaving no other factors significantly associated with heat risk. Western situation and low mean summer temperatures were associated with higher relative risks. We split latitude and mean temperature in two levels, below and above their respective median values of -3.4° (running slightly East of Madrid) and 23 C. The cross-tabulation of both defined four groups that roughly identify the climatic zones in the Iberian Peninsula (Figure 4): Oceanic climate (RR = 1.57: 95%CI = [1.39-1.79]), Semiarid Mediterranean climate (RR = 1.27: 95%CI = [1.19-1.34]), Continental Mediterranean (RR = 1.29: 95%CI = [1.23-1.36]), and Mediterranean climate (RR = 1.12: 95%CI = [1.04-1.20]).

## Discussion

Our results confirm that extreme heat days have a clear impact on total mortality in Spain. This has also been found in other multi-city European Studies [12,15,16], even in temperate climates [17]. To our knowledge, this is the first study using official thresholds defined by a heat health warning system.

These have been mainly based on the 95^th ^percentile (though with modifications in some scenarios, as previously described) of the 20-years historic series of minimum and maximum daily summer temperatures in each capital city. However, this criterion was only previously associated with health effects in the largest cities of Madrid [5], Barcelona [6] and Seville [4], whereas in others was not validated. Although the number of excesses was relatively homogeneous between the provincial capital cities in each Autonomous Region, there were substantial differences between regions. There were also a few with a surprisingly lower or higher than expected in comparison to other cities of the same Region.

There are several reasons that cities might show atypical relative risks during the supra-threshold days. Firstly, the official thresholds may not be a good indicator, for that city, of days at high risk. Specifically, the 95^th ^percentile may not be a good indicator of risk or the exceptions made to the 95^th ^percentile in the official thresholds criteria were inadvisable. Some of the cities with unusually high risks do appear to have been such exceptions. For example, Oviedo (RR = 2.08, 95%CI = [1.30-3.31]) had both the minimum and maximum threshold revised upwards to their *'floor' *levels of 20 C and 33 C, and Zamora (RR = 2.70, 95%CI = [1.50-4.86]) had a minimum temperature threshold (22 C) appreciably higher that the 95^th ^percentile (18.6 C), indicating that the special rule for continental climates had operated. More generally, the meta-regression analysis showed that low mean summer temperature, which was where the exception rules applied, was a strong predictor for high relative risks. Together, these suggest that the upwards revision of thresholds in cooler cities lower than would be appropriate if the intention was to identify days which the same excess risk as other cities on supra-threshold days.

To shed further light on performance of the official thresholds in identifying days of greatest heat-related events, we compared the percentage increase in risk of death on extreme heat days in 2003, calculated in our study (results not shown) with a previous study, by Martinez-Navarro et al. [18], where observed mortality for the summer of 2003 (from 1st June to 31st August) was compared with the mortality that would have been expected on the basis of historic series since 1990. We found a very weak correlation (r = 0.09) between estimates of both studies, perchance due to imprecision in the two estimates. However, if we only consider the largest cites (over 400,000 inhabitants) this correlation becomes considerably larger (r = 0.59). Indicating that in all these the original thresholds given by 95^th ^percentiles were not revised upwards.

Identifying heat-health thresholds is a practical decision that should respond to credibility, precision and cost criteria [19]. In this study we have not sought to explore all aspects of this nor review all the ways this can be done, so that we retain focus on the simple question of whether the official thresholds have been successful in identifying, in each provincial capital city, days with elevated risks of mortality and whether extent of elevation was about the same level across cities. For the same reason we did not explore factors that might modify the extreme heat impact, such period of time in the summer and duration of the extreme heat events, nor did we compare extremely hot to all other days, as done in some studies. Therefore our estimates are not entirely comparable with other published studies that quantified health effects of extreme hot temperatures.

The geographical variability in the distribution of the extreme heat-mortality effects roughly identifies the climatic zones in the Iberian Peninsula. This could partially be explained by the fact that city-specific risk estimates are calculated using official thresholds, which are at different percentiles of the temperature distribution, which may have added 'artificial' heterogeneity between climatic zones. However, when we used a fixed 95% percentile thresholds heterogeneity in relative risks did not differ substantially (I^2 ^= 51.4% vs. 54.9%). Thus there may still be a case for different thresholds for provincial capital cities based on their climatic region. Effects of high daily temperatures on mortality in English regions have been predicted from the region's climate at the 93 rd percentile of summer mean temperature [17]. Similarly, regional thresholds have been defined for heat-health warning systems in France [20] and Australia [21]. Age, urban environment and low socio-economic level have been identified as risk factors associated with heat-related mortality [22,23].

A major strength of the current study was the availability of long time series data sets from all provincial capital cities of Spain, providing enough power to identify and estimate extreme heat effects in all climatic regions of Spain. This study also includes the 2003 summer, which was an unusually hot summer in Europe. For this reason, we tested the sensitivity of our results by removing 2003 from the analysis. However, the overall risk estimate did not differ substantially (RR = 1.21; 95%CI = [1.16-1.27]). This may be explained by the fact that 1996 summer also recorded similar high temperatures in Spain, especially in Central Regions. Overall risk estimate for 1996 (RR = 1.29; 95%CI = [1.08-1.53]) was close to 2003 (RR = 1.31; 95%CI = [1.22-1.40]). Therefore, in a 10 years period we found another extremely hot summer, besides 2003, with a high impact on mortality. This agrees with the latest report issued by the Intergovernmental Panel for Climate Change indicates that climate change will lead to an increase in the frequency and intensity of heat waves [24]. Predictions for the Iberian Peninsula, using general circulation models, indicate a uniform increase in temperature over the course of the 21st century, with an average upward trend every 10 years between 0.6 C to 0.7 C in summer, a greater number and higher frequency of days with extreme heat temperatures in summer [25].

However, ecological time-series studies can be affected by various biases, in particular potential confounding due temporal patterns. In this study well-established methods were used to control for trend and seasonality. Additionally, some air pollutant levels, which are higher in summer in many European areas [2], are associated with an increase in mortality and may be associated with hot temperatures as well. In our case, it is difficult to ascertain whether the triggering factor for death in extreme heat days is exclusively due to an increase in temperature or to an increase of air pollution levels that tend to accompany such high temperatures. Unfortunately, due to lack of data our study was unable to assess the influence of air pollutants and other weather variables, such as humidity (although there is little evidence that it is associated with mortality) [11].

Although official thresholds gave consistent relative risk in the large capital cities, mainly because from previous studies [4-7] the 95^th ^percentile seems to be a 'natural' threshold of triggering mortality for heat days, in some other cities heat thresholds should be updated to better represent impact on health. In this sense, a very recent study conducted in Castilla La Mancha region showed how these 'natural' heat thresholds for maximum temperature range from the 92^nd ^percentile in Cuenca up to 97th percentile in Albacete and Toledo [26]. Therefore, criterion for the choice of thresholds must be based on epidemiological instead only on meteorological basis. In this sense, further research needs to be done to describe and understand how is the relationship of heat and mortality in these cities.

## Conclusions

This study confirmed that extreme heat days, as defined by the official thresholds of the *"National plan for preventive actions against the effects of excess temperatures on health"*, have a considerable impact on total daily mortality in Spain. However, relative risks on above-threshold days varied across the climatic zones of the Iberian Peninsula being higher in Northern cities with cooler climates, probably because thresholds were reset to values above the original 95^th ^percentiles in these cities. These should be updated to identify days of equally high risk.

## Competing interests

The authors declare that they have no competing interests.

## Authors' contributions

AT, IZ, CL, and JD conceived the study. AT analyzed the data in consultation with BA, and AG. AT, and IZ wrote the draft version and revisions of the manuscript according to the contribution of BA, AG, CL, and JD. All authors read and approved the final version of the manuscript.

## Pre-publication history

The pre-publication history for this paper can be accessed here:

http://www.biomedcentral.com/1471-2458/12/133/prepub

## Supplementary Material

Additional file 1 **Map of Spain by administrative divisions (Autonomous Regions and Provinces)**. Click here for file

Additional file 2 **Geographic and socio-economic characteristics of capital cities in Spain**. Click here for file
