# Supplementary figures and images for: Mortality on extreme heat days using official thresholds in Spain: a multi-city time series analysis
Source: BMC Public Health. 2012 Feb 17;12:133. doi: 10.1186/1471-2458-12-133 (PMC3314548; doi:10.1186/1471-2458-12-133)

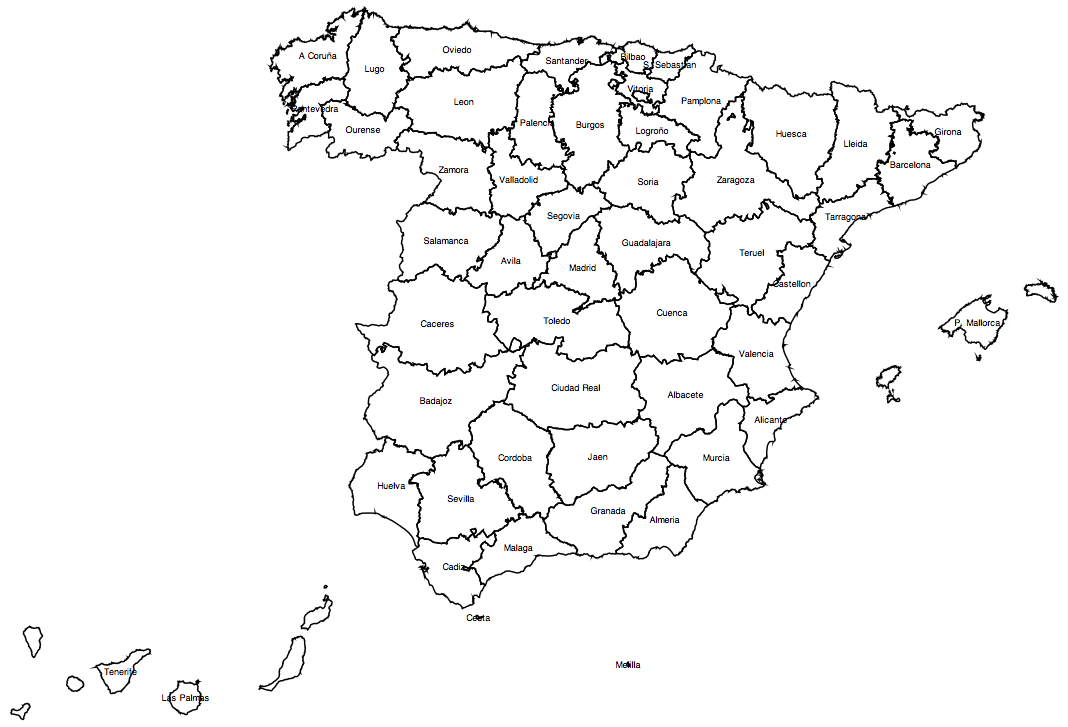

Supplement: Additional file 1 — Map of Spain by administrative divisions (Autonomous Regions and Provinces). [file 1471-2458-12-133-S1.JPEG]
