# Supplementary material for: Mortality on extreme heat days using official thresholds in Spain: a multi-city time series analysis
Source: BMC Public Health. 2012 Feb 17;12:133. doi: 10.1186/1471-2458-12-133 (PMC3314548; doi:10.1186/1471-2458-12-133)
Supplement: Additional file 2 — Geographic and socio-economic characteristics of capital cities in Spain. [file 1471-2458-12-133-S2.PDF]

Autonomous Region	City (capital)	Coordinates Latitude	Longitude	Altitude (m)	Surface (km2)	Population* Total	>65 years	Per capita income**
**Andalucía**	**Almería**	-2.27	36.49	23	296.2	169,027	12.9%	10,239 €
	**Cádiz**	-6.17	36.31	11	12.3	142,449	14.2%	7,750 €
	**Córdoba**	-4.46	37.52	120	1255.2	311,708	14.1%	7,909 €
	**Granada**	-3.36	37.10	738	88.0	244,767	16.3%	7,731 €
	**Huelva**	-6.57	37.15	54	151.3	140,583	12.7%	8,508 €
	**Jaén**	-3.47	37.45	573	424.3	109,247	13.7%	7,802 €
	**Málaga**	-4.24	36.43	8	395.1	530,553	13.6%	8,007 €
	**Sevilla**	-5.59	37.23	7	141.3	701,927	14.7%	7,997 €
**Aragón**	**Huesca**	-0.24	42.08	488	161.0	45,627	18.7%	11,380 €
	**Teruel**	-1.06	40.20	915	440.4	30,047	19.3%	10,660 €
	**Zaragoza**	-0.52	41.39	199	1063.1	603,367	17.7%	11,076 €
**Asturias**	**Oviedo**	-5.50	43.21	127	186.7	200,453	18.7%	9,616 €
**Baleares**	**P. Mallorca**	2.39	39.34	13	208.6	326,993	14.7%	12,503 €
**Canarias**	**Las Palmas**	-15.24	28.05	23	100.6	354,757	12.2%	9,798 €
	**Tenerife**	-16.14	28.27	4	150.6	213,050	13.5%	9,359 €
**Cantabria**	**Santander**	-3.48	43.27	15	34.8	184,165	18.7%	10,231 €
**Castilla La Mancha**	**Albacete**	-1.51	38.59	686	1125.9	147,527	13.0%	8,478 €
	**Ciudad Real**	-3.55	38.59	628	285.0	61,138	14.7%	8,837 €
	**Cuenca**	-2.07	40.04	920	911.1	45,100	17.7%	9,165 €
	**Guadalajara**	-3.09	40.38	708	235.5	69,959	14.3%	8,636 €
	**Toledo**	-4.01	39.51	454	231.8	67,617	14.2%	8,511 €
**Castilla y León**	**Ávila**	-4.41	40.39	1128	230.7	47,682	16.4%	9,148 €
	**Burgos**	-3.42	42.20	856	107.1	162,802	16.3%	11,288 €
	**León**	-5.34	42.35	838	39.0	139,809	19.4%	9,152 €
	**Palencia**	-4.32	42.01	749	94.9	80,332	17,7%	10,315 €
	**Salamanca**	-5.40	40.57	802	39.3	158,720	18.7%	9,092 €
	**Segovia**	-4.07	40.57	1002	163.6	54,175	18.1%	10,799 €
	**Soria**	-2.28	41.46	1063	271.8	34,045	19.0%	11,270 €
	**Valladolid**	-4.43	41.39	691	197.5	319,998	15.2%	10,561 €
	**Zamora**	-5.45	41.29	652	149.3	64,906	18.1%	8,729 €
**Catalunya**	**Barcelona**	2.10	41.23	12	98.2	1,503,451	22.0%	11,708 €
	**Girona**	2.49	41.58	76	39.0	72,682	15.6%	13,076 €
	**Lleida**	0.37	41.37	155	212.0	112,207	16.9%	13,058 €
	**Tarragona**	1.22	41.15	69	62.4	113,016	15.3%	11,923 €
**Ceuta y Melilla**	**Ceuta**	-5.18	35.53	27	19,5	73,704	10.5%	9,434 €
	**Melilla**	-2.33	35.27	48	13.4	56,929	11.2%	9,481 €
**Extremadura**	**Badajoz**	-6.58	38.52	185	1470.4	136,613	12.3%	7,545 €
	**Cáceres**	-6.22	39.28	459	1750.3	78,614	13.5%	7,539 €
**Galicia**	**A Coruña**	-8.23	43.22	0	37.8	243,402	17.1%	8,518 €
	**Lugo**	-7.33	43.00	465	329.8	87,480	18.3%	8,737 €
	**Ourense**	-7.51	42.20	132	84.6	109,120	17.7%	9,009 €
	**Pontevedra**	-8.38	42.26	77	118.3	74,139	14.8%	9,057 €
**La Rioja**	**Logroño**	-2.26	42.27	384	79.6	127,093	16.1%	11,943 €
**Madrid**	**Madrid**	-3.41	40.24	667	605.8	2,879,052	19.4%	12,781 €
**Murcia**	**Murcia**	-1.07	37.59	43	886.0	353,504	13.8%	8,736 €
**Navarra**	**Pamplona**	-1.38	42.49	449	25.2	180,483	17.5%	12,900 €
**País Vasco**	**Bilbao**	-2.55	43.15	19	41.3	357,589	19.6%	12,473 €
	**San Sebastián**	-1.58	43.19	6	60.9	179,208	18.9%	12,788 €
	**Vitoria**	-2.40	42.50	525	276.8	217,154	14.1%	12,785 €
**Valencia**	**Alicante**	-0.28	38.20	0	201.3	272,432	16.2%	9,697 €
	**Castellón**	-0.12	39.59	30	108.8	139,712	15.0%	11,256 €
	**Valencia**	-0.22	39.28	15	134.6	739,412	17.3%	10,082 €

* Source: Instituto Nacional de Estadística, Padrón Municipal del año 1999.
** Source: Instituto Nacional de Estadística, Contabilidad Regional de España. Base 2000.
